# First person – Shannon Taylor

**DOI:** 10.1242/bio.047860

**Published:** 2019-09-15

## Abstract

First Person is a series of interviews with the first authors of a selection of papers published in Biology Open, helping early-career researchers promote themselves alongside their papers. Shannon Taylor is first author on ‘The *torso-like* gene functions to maintain the structure of the vitelline membrane in *Nasonia vitripennis*, implying its co-option into *Drosophila* axis formation’, published in BiO. Shannon is a Master's student in the lab of Peter Dearden at the University of Otago, Dunedin, New Zealand, investigating evolution and development (EvoDevo) and the philosophy of science, thus far using *Nasonia* as a model species to study various EvoDevo questions.


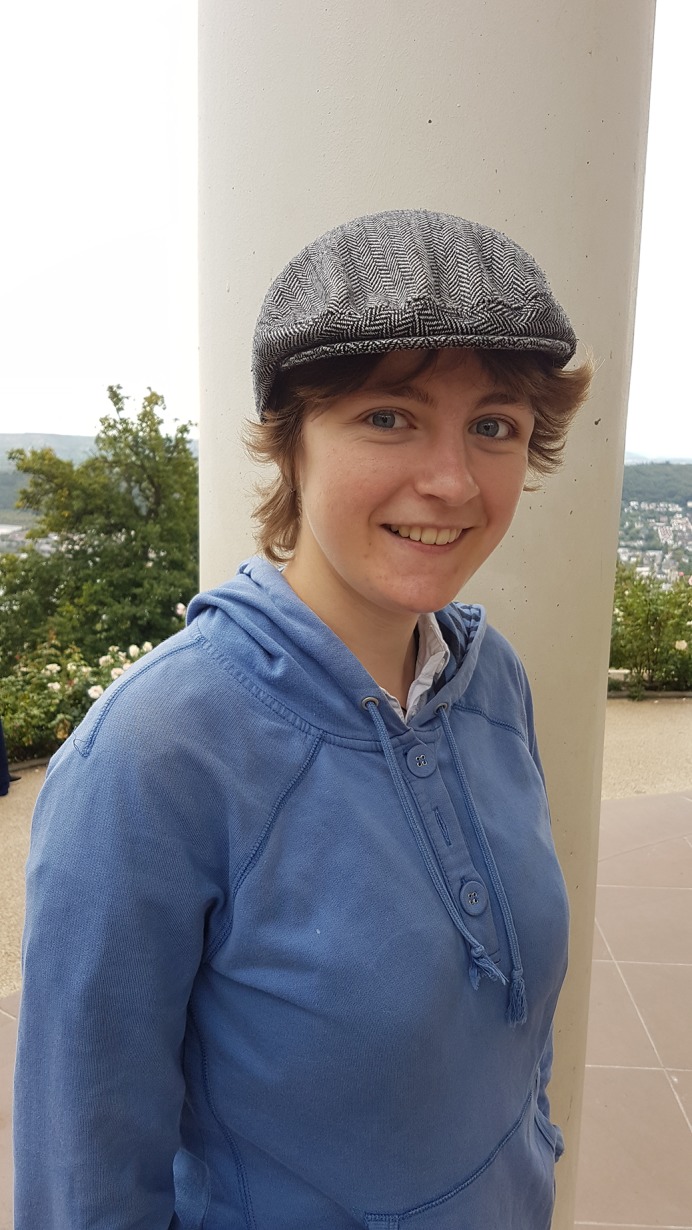


**Shannon Taylor**

**What is your scientific background and the general focus of your lab?**

My undergraduate degree is in biochemistry, but as I've been working in an EvoDevo lab since early in my degree, I'm as much a geneticist as a biochemist! My research background is primarily classic EvoDevo work on *Nasonia*.

Peter Dearden's lab focuses on the evolution of developmental pathways: how does evolution alter developmental processes to produce diverse forms? One pathway we are particularly interested in is the *Drosophila* terminal patterning pathway, which has a unique and complex evolutionary history. We have also done a lot of work on honeybees, both their development and plastic events in their life cycle, and are now working on the genetic basis of parasitoid resistance.

“How does evolution alter developmental processes to produce diverse forms?”

**How would you explain the main findings of your paper to non-scientific family and friends?**

How genetic processes control development is a fundamental and fascinating question in biology. Though we have a very thorough understanding of fruit fly embryonic development from decades of work, it is unclear how well this knowledge transfers to other species. For example, fruit flies specify their termini using a set of genes that are not all present in many other insects, particularly bees, ants and wasps. This raises an important question: what are these genes doing when not specifying termini? Consistent with previous genetic work, we found that most of these genes are not required for early wasp (*Nasonia*) development. Interestingly, however, one gene (*torso-like*) was required for eggshell integrity, a distinct functional role from the one it has in the fruit fly, despite sharing similar biochemical activity. This illustrates the fact that the same gene can have vastly different roles over evolutionary time!

**What are the potential implications of these results for your field of research?**

Our work showed that *torso-like*, a gene required for cell signaling and embryonic development in *Drosophila*, is instead required for vitelline membrane (part of the insect eggshell) integrity in *Nasonia*. As the *Drosophila* vitelline membrane is known to carry important positional information for embryogenesis, and is required for gastrulation, the finding that a ‘developmental’ gene is required for eggshell integrity underscores the tight link between development and this structure.

More broadly, this work points to the importance of extracellular structures and the biochemical functions of proteins in development. Development doesn't just require networks of genes dictating cell fate: tissue mechanics, morphology and non-transcription factor genes are also critically important.

**Figure:**
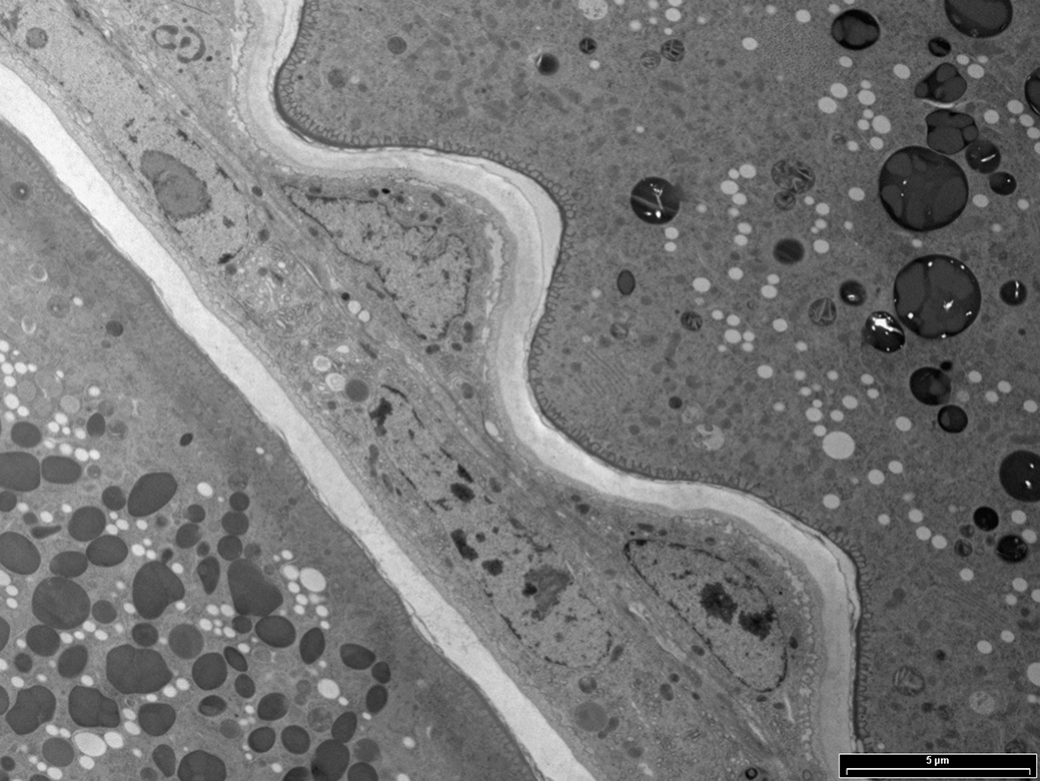
**Electron microscopy micrograph of two *Nasonia* oocytes (left and right), separated by developing eggshell and follicle cells (center).**

**What has surprised you the most while conducting your research?**

Before I started research, I bought into the ‘lone genius’ myth – that science is done by sole geniuses. According to this account, science is done alone, and you have to be a genius to make a meaningful contribution to science. This is wrong! Science in the 21st century is a collaborative, social process, for better and for worse. I would not have been able to complete this project without the fantastic mentoring I've received over the years, and the paper itself is a collaboration between scientists across two countries, some of whom I've never met face-to-face! Of course, the downside of this is that our human biases often creep into the work we do.

“Science in the 21st century is a collaborative, social process, for better and for worse.”

**What, in your opinion, are some of the greatest achievements in your field and how has this influenced your research?**

Technical advances allowing us to do robust genetics in non-traditional model species have vastly expanded the types of EvoDevo biology we can do. RNA interference (RNAi) and more recently, CRISPR, have allowed us reliably knock down any gene in virtually any species, enabling robust comparison of gene functions across evolutionary time. This paper simply would not exist without RNAi! The genomics era has also made it possible to investigate the conservation of genes and genetic pathways. More recently, new methods for visualizing gene expression, like hybridization chain reaction, are enabling us to quickly and easily obtain comparative gene expression patterns. The ability to easily perform multi-gene co-expression analysis really opens up new avenues of research, and is proving invaluable for my Master's (studying the *Nasonia* segmentation network). Such technical advances expand the types of questions we can answer in EvoDevo.

**What changes do you think could improve the professional lives of early-career scientists?**

I wish I had easy answers… at the moment I and many of my peers are struggling with the instability of science. I don't know what country I'll be living in in 12 months (for my PhD) and that's really scary. Exciting, but scary. Because I'm from New Zealand, where there are not many people doing the types of research I'm interested in, I do know that I'll probably be 13,000 km from home. In some ways this is a good problem to have; it means science is very international, and I'm extremely lucky to have these options. But I wish it didn't feel like a choice between science and home/family.

**What's next for you?**

I've become very interested in the ways models can enrich and extend our understanding of biology, both from an empirical and philosophical viewpoint. At the moment I'm working towards a Master's in genetics and philosophy of science, using *Nasonia* segmentation as a ‘model system’ in which to answer these questions. Next I hope to start a PhD investigating similar questions, though probably in a new system.

**Where are you now?**

I'm currently on holiday, cycling (and catching the train) up the Rhein river!
